# Ourselves

**Published:** 1881-12

**Authors:** 


OURSELVES.—With this number the second volume of the INDEPENDENT PRACTITIONER is brought to a close. We call attention to the large number of valuable articles, as shown by the index in this number, which have appeared during the current year. We shall not rest upon what we have done in the past, however, but make a long step in advance in the next volume. We have secured promises of contributions from a large number of the most eminent men in the profession, among whom we may mention, at present, Professors Alfred L. Loomis, Wm. A. Hammond and Fessenden N. Otis, of this city; James E. Garretson and Roberts Bartholow, of Philadelphia; Chas. F. Bevan, of Baltimore, and F. Peyre Porcher, of Charleston.

Attention is also called to the circular giving our clubbing rates, mailed with this number of the journal. By ordering books and journals through us a considerable sum may be saved, where more than one journal is taken regularly. We ask our present and all intending subscribers to send in their names and subscriptions at once, in order that we may know how large an edition to print of the January number, which begins volume III. Specimen copies will be sent on application.

